# Beyond the Individual -A Scoping Review and Bibliometric Mapping of Ecological Determinants of Eating Behavior in Older Adults

**DOI:** 10.3389/phrs.2022.1604967

**Published:** 2022-08-03

**Authors:** Íris Rafaela Montez De Sousa, Ina Bergheim, Christine Brombach

**Affiliations:** ^1^ Institute of Food and Beverage Innovation, Life Sciences and Facility Management, Zurich University of Applied Sciences, Wädenswil, Switzerland; ^2^ Molecular Nutritional Science, Department of Nutritional Sciences, University of Vienna, Vienna, Austria

**Keywords:** food environment, ecological model, bibliometric analysis, eating behavior, community-dwelling elderly, nutrition communication

## Abstract

**Objectives:** The objective of this scoping review was to summarize and provide a visual overview of the present-day knowledge on ecological determinants of eating behavior in community-dwelling elderly persons in relation with nutrition communication, considering the evolution of the field. The second objective was to integrate results in recommendations for the development of nutrition communication strategies.

**Methods:** A literature review was performed on Medline, PubMed and Google Scholar, according with the PRISMA protocol for scoping reviews. An *a-priori* analysis was executed by categorizing determinants from the literature according with the different levels represented in the ecological framework and an *a-posteriori* analysis by using VosViewer for a chronological bibliometric mapping analysis.

**Results:** Of 4029 articles retrieved, 77 were selected for analysis. Initial publications focused more on individual determinants of eating behavior. Over time, there was a shift towards a holistic view of eating behavior considering the “food environment”, including social networks, physical settings and public policy.

**Conclusion:** Beyond the individual, all ecological levels are relevant when targeting eating behavior in the elderly. Nutrition communication strategies should be structured considering these influences.

## Introduction

According to the European Commission, by 2060 the percentage of individuals over 65 years will have risen from 19.3% (in 2016) to about 29.0% [1]. This shift is thought to have tremendous impacts on society [1, 2]. Although life expectancy has increased, time of “healthy life” is not increasing to a similar extent. Older adults face various health issues that may be improved if “structural, economic and social drivers” of health are addressed [3]. To date, measures taken to tackle these often fail. A better understanding of the health challenges associated with aging may improve the odds for good health and life quality in the elderly [1].

One of the major determinants of healthy aging is nutrition [4, 5]. Epidemiological studies suggest that maintaining a healthy diet leads to a decrease in morbidity specially related to cognitive decline and metabolic diseases, which also translates to reduced healthcare costs [6, 7]. These findings reinforce the importance of creating adequate nutrition interventions that lead the elderly to adjust their food choices so that their cognitive and overall health is maintained, or even improves [8].

One of the main challenges for healthcare providers, scientists and policy makers is to develop communication strategies to successfully convey scientific information to older adults resulting in sustainable behavioral changes [9]. To create successful evidence-based nutrition communication strategies, it is important understand how various internal and external determinants influence older adults’ food choice [10]. Findings of several studies suggest that eating behavior results from a complex interaction of factors not only directly related to the individual but also the contextual environment [11].

The contextual environment and its interaction with the individual are well portrayed in the “Ecological Framework”, a conceptual model composed of “spheres” that have an influence on human behaviors [12]. These spheres include individual, social, physical and macro level environments [12]. Furthermore, ecological determinants, and their interactions, that influence eating behavior in older adults need to be considered, while acknowledging that these are not static but may vary over time [13, 14]. Generally, this model is thought to be adequate for achieving a holistic understanding of eating behavior, thereby also laying the basis to the development of promising communication strategies focused on healthy eating [15].

Several studies investigated determinants of eating behavior in the elderly [4, 16–19]; however, study designs are heterogeneous both in methodology and determinants explored. There is a lack of an overview of the research performed. Undertaking a scoping review to provide a “map” of the body of literature in this field of research will be valuable to identify key findings and gaps for further investigation.

The objective of this scoping review with bibliometric mapping was to summarize and provide a visual overview about what is known regarding ecological determinants of eating behavior and food choice in older adults in relation with nutrition communication possibilities. The evolution of the field over time was considered as well. The second aim was to provide the basis for the development of novel, improved nutrition communication strategies targeting eating behavior in older adults.

There was a focus on literature after 2000 because the aging of the world population is a major trend of this period. Besides, during this century there was a huge advance in the use of digital technologies, which also affected food environments [20].

There is a need for an overview of what determines eating behavior in the elderly and how these determinants can be modulated towards healthier eating habits [21]. While the topic has been reviewed in some publications, the evolution of the field over time has not been studied so far. Besides, the subject has not been explored by using bibliometric mapping and the Ecological Framework [16, 21, 22].

## Methods

### Literature Search

This scoping review was performed in accordance with the PRISMA-ScR Checklist and the framework for scoping reviews proposed by Arksey and O’Malley [23, 24]. Even though an internal review protocol was elaborated this was not registered in any open platform. The main research question was: What is known, from literature published from 2000 onwards, about ecological determinants of eating behavior in older adults that are important to consider for developing nutrition communication strategies?

The databases used for the search were Google Scholar, Medline (*via* ProQuest) and PubMed. The search expression used was: (elderly OR senior OR older OR age) AND (nutrition OR food OR eating OR meals OR feeding OR “eating behavior”) AND (communication OR education OR “health promotion” OR program) AND (determinants OR factors OR influence) NOT (child OR teenager OR adolescent OR student OR young). These terms were searched exclusively in the title of publications in Google Scholar, in the title and abstract in PubMed and anywhere except the full text in Medline. The ideal approach was to search in all databases “anywhere except full text”, however this was not possible. When possible, filters were applied so that only studies pertaining to humans, older than 65 years old were obtained. The age filter was added to reduce the number of hits obtained in the search to a feasible amount to analyze. The date of the last search was the 10th of June of 2021.

Firstly, duplicate articles were removed; afterwards the title and abstracts were examined, and articles selected according to the criteria for inclusion (Table 1) and exclusion (Table 2). The full text was read for the remaining articles to assess whether these fitted the criteria. If the full text was not available, the articles were excluded. Intervention studies were excluded because the goal was to obtain an overview of determinants of eating behavior in older adults’-built environment and not yet to explore how interventions targeted these. Selected publications were summarized according to the author(s), year of publication, origin, purpose, study characteristics, outcome measurements and results.

**TABLE 1:** Inclusion Criteria (scoping review, high-income countries, 2000–2020).

Language	Setting/Location	Publication period	Age of participants	Topic	Population characteristics
English, Portuguese, Spanish, Italian, German, French or Dutch	High-income countries	2000–2020	According to the definition of each publication (the youngest age referred as older adult in this review is 50 years old)	Exploring determinants of eating behavior/food choice/eating habits in older adults	Community-dwelling older adults, independent for most daily activities

**TABLE 2:** Exclusion Criteria (scoping review, high-income countries, 2000–2020).

Setting/Location	Publication period	Topic	Population characteristics	Type of study
Low-income countries	Before 2000 or after 2020	Studies exclusively researching malnutrition, obesity, or physiological effects of nutrition in older adults	Institutionalized, home or bed-bound older adults	Intervention studies (including meal delivery programs)

### *A-Priori* and *A-Posteriori* Analyses

*The a-priori* analysis consisted of qualitatively extracting the ecological determinants of eating behavior mentioned in the literature by reading the publications. These determinants were grouped in categories and assigned to the levels of the Ecological Framework proposed by Story et al. [12]. Categories mentioned repeatedly in literature were given bigger font sizes than the ones mentioned less often.

The software VosViewer was used for the *a-posteriori* analysis [25]. The software identified frequent terms among literature, these were considered to be the ones present in the title and abstract of the publications at least five times. As for the relevance score, no cut-off points were defined, the highest relevance scores obtained after the analysis were reported [25]. To analyze the evolution of research on this topic, publications were divided in four chronological periods: 2000–2004, 2005–2009, 2010–2014 and 2015–2020.

## Results

**FIGURE 1:**
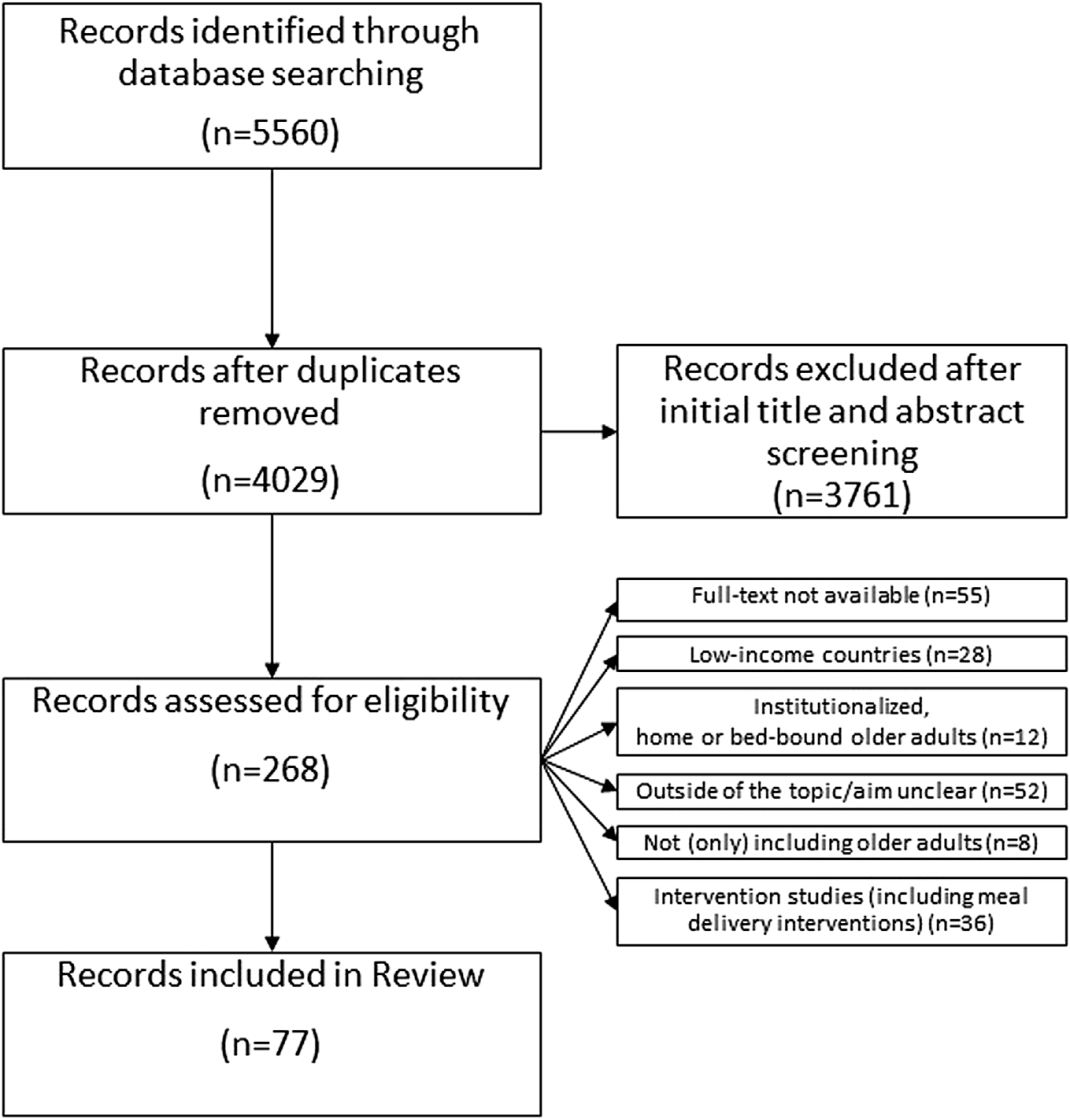
Flow-chart of the article selection process (scoping review, high-income countries, 2000–2020).

**FIGURE 2:**
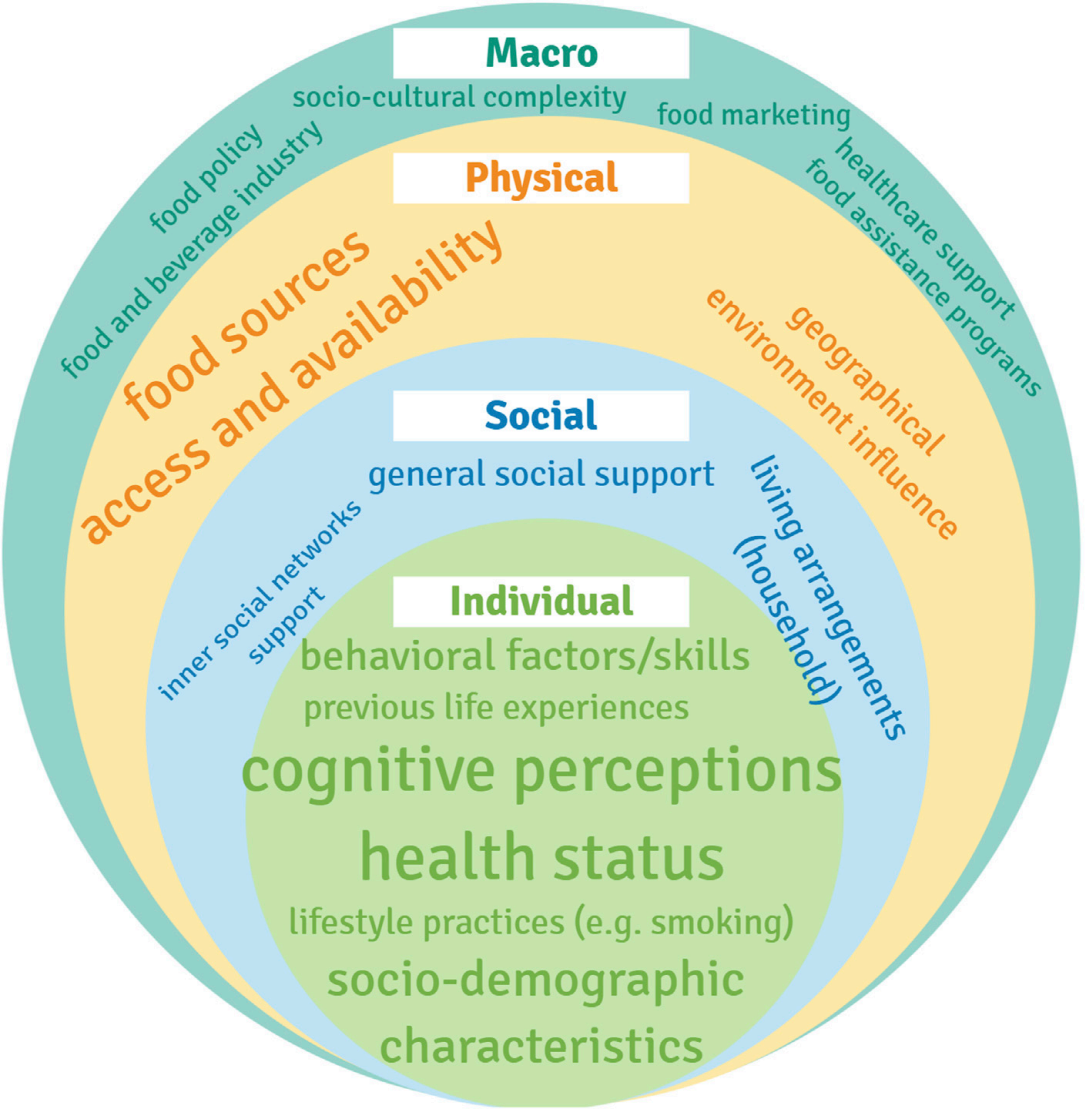
Ecological model adapted from the ecological framework proposed by Story et al. [12], with categories of determinants according with the literature analyzed (scoping review, high-income countries, 2000–2020).

A total of 5,560 publications were identified in the search, which were reduced to 4,029 after removing duplicates. After screening the title and abstracts, there were 268 articles left. These were accessed for eligibility by reading the full text, 77 publications were selected for analysis (flow-chart in Figure 1). Despite of the use of the age filter, insightful studies with participants younger than 65 years were obtained. Hence an age range of 50 years or more was applied considering different definitions of older adult [21]. A summary of the publications selected can be found in Table 3. In Figure 2 is shown the Ecological Framework [12] with the categories of ecological determinants found in the literature and font sizes indicating more and less common categories. A more detailed overview of how determinants of eating behavior were grouped in categories can be found in the Supplementary Material (Ecological determinants of eating behavior in elderly grouped in categories, per period).

**TABLE 3:** Data Charting of Selected Articles (scoping review, high-income countries, 2000–2020).

Author, Year, Origin	Purpose of the study	Study characteristics	Outcome measurements	Main results
McDonald et al. [26] United States	Specify strategies of nutritional self-management of widowed older men in rural areas and identifying factors putting them at nutritional risk.	Design: qualitative	In-depth semi-structured interviews over the period of 1 year (topics: background history, health status, food security, self-care and (in)formal support), dietary intake data from FFQ, instrumental activities of daily living and self-rated health scales	Nutrition strategies were divided in three tasks: acquiring food, using food and maintaining food security. The strategies that successfully helped to accomplish these tasks were self-care, formal and informal support. Various resources that enabled these strategies were identified.
		Setting: US (rural areas)		
		Subjects:12 older men widowers		
		Age range: ≥70 years		
Van Rossum et al. [38] Netherlands	Define differences in dietary intake in older adults according to their SES (education).	Design: cross-sectional	Dietary intake data from semiquantitative FFQ	Differences between groups were small. Nevertheless, generally lower educated participants had a higher intake of most macronutrients comparing with the higher educated participants.
		Setting: Netherlands (The Rotterdam Study)		
		Subjects: 5406 older adults		
		Age range: ≥55 years		
De Almeida et al. [27] Portugal	Identify attitudes towards food in the European elderly.	Design: cross-sectional	Face-to-face interview-assisted questionnaires about food choices, barriers to healthy eating and other perceptions of healthy eating	“Quality and freshness,” “trying to eat healthily” and “price” were reported as the most important determinants of food choice. Healthy eating was defined as “less fat,” “more fresh vegetables and fruit” and “natural foods.” “Staying healthy,” “preventing diseases” and “promoting quality of life” were the main reasons to follow a healthy diet. “Self-control,” “resistance to change” and “price” were the main barriers.
		Setting: Europe		
		Subjects: 1843 older European citizens		
		Age range: ≥65 years		
Haveman-Nies et al. [37] Netherlands and United States	Evaluate the diet quality of European and American elderly using dietary patterns.	Design: cross-sectional	Food intake data summarized into clusters and dietary scores	Dietary intake varied across geographical regions. Elderly from Southern Europe and the US (Framingham) had higher dietary quality than Northern Europeans.
		Setting: Europe and US		
		Subjects: 828 older adults from the US and 1282 older adults from Europe		
		Age range: 70–77 years		
Pryer et al. [31] United Kingdom	Identify groups of elderly in the United Kingdom that report similar dietary patterns.	Design: cross-sectional	4-day weighed dietary records resulting in food groups and nutrient consumption; socio-economic, demographic, and behavioral data.	There were differences in socio-economic and behavioral characteristics within dietary clusters. Elderly following a traditional and mixed diet were more likely to be smokers, come from manual job classes, have a lower income and education, and a higher age.
		Setting: United Kingdom		
		Subjects: 1097 older adults		
		Age range: ≥65 years		
De Castro [41] United States	Investigate age-related changes in eating behavior.	Design: cross-sectional	Dietary nutrient intake from 7-day dietary records; Demographic data; Data about dietary restraint, disinhibition and hunger.	Elderly ate with fewer people and were less responsive than the younger participants to self-reported hunger. Elders were as responsive as the youngest to social facilitation, palatability, cognitive restraint, time and location of the meals.
		Setting: US		
		Subjects: 46 older adults		
		Age range: ≥65 years (younger groups were included and compared)		
Österberg et al. [32] Sweden	Investigate the relationship between masticatory ability and nutrient, energy, and food intakes.	Design: cross-sectional	Dental examinations, self-assessed masticatory ability, and dietary interviews	Impaired health and dentition were associated with masticatory problems, yet the correlation between dental status/bite force and dietary intake was generally weak.
		Setting: Sweden		
		Subjects: 160 older adults		
		Age range: 80 years		
Tucker and Reicks [30] United States	Determine if physical exercise is a potential “gateway behavior” for healthy eating in elderly.	Design: cross-sectional	Perceived benefits and barriers, self-efficacy, and stages of change regarding physical exercise and eating behavior	According with the Transtheoretical model, elderly in later stages of change for physical exercise were also more likely to be in later stages for fruit and dairy consumption. The same was not found for vegetables consumption and fat avoidance.
		Setting: US (large Midwestern city)		
		Subjects: 205 older adults		
		Age range: ≥65 years		
Vitolins et al. [33] United States	Evaluate the intake of different food groups of African American, European American, and Native American elderly living in two rural communities.	Design: cross-sectional	Demographic, personal and food intake data resulting from an interviewer-administered semiquantitative FFQ	African Americans and Native Americans consumed fewer portions of various food groups (meat, fruit, vegetables, fats, oils and sweets) compared to the European Americans. Participants over 85 years old were the biggest consumers of fat, oils and sweets.
		Setting: US (two rural communities in central North Carolina)		
		Subjects: 130 older adults		
		Age range: >70 years		
Callen and Wells [34] United States	Explore barriers and opportunities to maintain the nutritional health of independent-living elderly, older than 80 years old.	Design: qualitative	Demographic data, risk scales, dietary intake (24 h diet diary), physical status data and data about barriers and opportunities to maintaining nutritional health from in-home interviews	Social connectedness was the major help in maintaining nutritional health and the major barriers were health problems. The ones perceiving more barriers had a higher probability of depression.
		Setting: (Midwestern) US		
		Subjects: 68 older adults		
		Age range: ≥80 years		
Corrêa Leite et al. [39] Italy	Describe the dietary patterns of Italian elderly and the determinants associated with dietary habits.	Design: cross-sectional	Dietary intake data from FFQ and socio-demographic data	Elderly from Northern Italy consumed higher amounts of animal fats, sugar and alcoholic beverages and Southern Italians had a higher consumption of fruit, vegetables, fish and olive oil.
		Setting: (Northern and Southern) Italy		
		Subjects: 2312 older adults		
		Age range: ≥65 years		
Johnson and Garcia [36] Canada	Examine the physical activity and diet of elderly immigrants and the factors that influence these.	Design: cross-sectional	Socio-demographic and personal data from background questionnaires; nutritional risk from nutrition screening tools; dietary intake data from 24-h dietary recalls, and physical activity profile from a questionnaire.	About 73% of the elderly were in moderate to high risk of malnutrition. The main problems were about food preparation, nutritional disease management and meeting nutritional needs.
		Setting: Canada		
		Subjects: 54 older adults from Cambodian, Latin-American, Vietnamese, and Polish groups		
		Age range: ≥62 years		
Kim et al. [28] United States	Explain elderly intention to consume dairy products and actual consumption using the Theory of Planned Behavior.	Design: cross-sectional	Dietary intake data from FFQ and attitudes, subjective norms, perceived behavioral control, and intention to consume dairy products measured from questionnaires	Attitude and perceived behavioral control contributed to predict the intention to consume dairy products, yet subjective norms did not have an influence.
		Setting: US (Community centers)		
		Subjects: 162 older adults		
		Age range: ≥65 years		
Palmer [35] United States	Discuss oral and nutritional changes that may occur with aging, review nutritional status and requirements, and propose strategies to address nutritional problems in elderly.	Design: review	—	Various factors may put the dental impaired elderly at risk for malnutrition: physiologic, oral, psychosocial, functional, and medical factors.
		Setting:		
		Subjects:		
		Age range:		
Rurik [40] Hungary	Obtain an overview of the lifestyle and nutrition habits of Hungarian elderly.	Design: cross-sectional	Questionnaire measuring socio-demographic factors, lifestyle factors, nutritional habits, meal patterns and food preferences, and anthropometric parameters	Hungarian older adults were generally overweight, lacked physical activity and had unhealthy nutritional habits.
		Setting: Hungary		
		Subjects: 250 older adults		
		Age range: men: ≥65 years; women: ≥60 years		
Sindler et al. [29] United States	Gather information from Holocaust survivors on how this experience influenced their attitude towards food.	Design: qualitative	Data about food-related memories from the Holocaust and liberation and how these influenced attitudes gathered from one-on-one semi structured interviews	Food attitudes were influenced by the Holocaust in five main ways: difficulty in throwing away food; storing food in excess; craving specific foods; difficulty to stand in line waiting for food and suffering anxiety when food is not readily accessible.
		Setting: US		
		Subjects: 25 older adults		
		Age range: 71–85 years		
Martin et al. [51] United States	Explore factors that influence dietary practices and patterns in low-weight elderly and nutritional advice received from healthcare providers.	Design: qualitative	Semi-structured interviews to explore the eating behavior of the participants	Eating alone, loneliness and other stressors were the main reasons for low weight.
		Setting: US		
		Subjects: 8 older women (followed by a healthcare practitioner during the past 6 months and with a BMI<24)		
		Age range: 65–91 years		
Payette and Shatenstein [16] Canada	Summarize the current knowledge and research gaps about determinants of healthy eating in Canadian elderly.	Design: review	Articles about determinants of healthy eating in seniors	Food choices in elderly are affected by health status and biological changes, yet these are mediated by socio-economic factors. Determinants of healthy eating are both individual and collective.
		Setting: Canada		
		Subjects:		
		Age range:		
Payne et al. [52] United States	Examine the association between vascular nutritional factors and depression.	Design: cross-sectional	Nutrient intake measured with FFQ, BMI and Keys score (measure of serum cholesterol raising capacity of diet)	The depression group had higher intakes of cholesterol and saturated fat, lower intake of alcohol and a higher Keys score than the comparison group.
		Setting: US		
		Subjects: 196 older adults divided in depression and comparison groups		
		Age range: ≥60 years		
Rurik [42] Hungary	Find differences in nutritional habits of older men and women.	Design: cross-sectional	Self-managed questionnaires about lifestyle and eating habits and FFQ	Meal frequency increased with aging. Women preferred lunch as the main meal and consumed more dairy, fruit, bread, biscuits, chocolate and coffee. Prices had a higher impact in food choice of women. Men consumed more often alcoholic beverages. Meat, eggs, and vegetables were preferred by men.
		Setting: Hungary		
		Subjects: 266 older adults		
		Age range: men: ≥65 years; women: ≥60 years		
Smith et al. [48] United States	Explore the culturally constructed meaning of salt, contrast the medical and cultural views, and discuss how this can be applied to health education and doctor-patient communication.	Design: qualitative	In-depth interviews about knowledge and beliefs about nutrients, nutrition related diseases and folk knowledge about disease prevention and nutrition and focus groups to identify and clarify themes from the interviews	Salt is seen as important for traditional foods but also as negative for medical issues. Older adults try to reconcile the importance of traditional foods for their identity with meeting medical recommendations.
		Setting: rural communities in the Southern US		
		Subjects: 116 ethnically diverse older adults		
		Age range: ≥60 years		
Walker et al. [50] United States	Examine the relationship of cognitive-perceptual determinants (based on the Health-Promotion Model) with healthy eating and physical activity.	Design: cross-sectional	Self-reported data about healthy eating and behavioral determinants from questionnaires, activity biomarkers and body weight	Higher self-efficacy, perceived benefits, and interpersonal support and fewer perceived barriers were positively associated with healthy eating and physical activity habits.
		Setting: US (rural areas)		
		Subjects: 179 older women		
		Age range: 50–69 years		
Giltay et al. [53] Netherlands	Explore if dispositional optimism is associated with healthy lifestyles and dietary habits.	Design: longitudinal	Four-item questionnaire to assess dispositional optimism, cross-check dietary history method, lifestyle factors questionnaire and BMI	Dispositional optimism was positively associated with physical activity, non-smoking, higher intakes of alcohol, fruit, vegetables, and whole-grain bread.
		Setting: Netherlands		
		Subjects: 773 older men		
		Age range: 64–84 years at start		
Shannon et al. [43] United States	Describe the diet quality of a cohort of elderly men and lifestyle determinants associated with poor diet quality.	Design: cross-sectional	Dietary intake data assessed with the modified Block FFQ; diet quality data measured with the Diet Quality Index-Revised and sociodemographic data	Diet quality was generally low. Important predictors of diet quality included age, total caloric intake, BMI, geographic residence, ethnicity, education, smoking and (not) using dietary supplements.
		Setting: US		
		Subjects: 5928 older men		
		Age range: 65–100 years		
Tannenbaum and Shatenstein [49] Canada	Recognize age differences influencing the level of exercise and healthy eating among Canadian older women and identify the interventions they would prefer to help them stay fit and eat healthily.	Design: cross-sectional	Survey about exercise frequency, nutritional risk, functional status, and factors that could facilitate the adoption and maintenance of physical activity and healthy dietary habits	About 35% of the younger women (<75 years), that did not exercise enough/did not eat healthily, desired suitable health promotion programs, low-cost and written materials, whereas older women preferred transportation to programs. Generally, participants at nutritional risk and sedentary mentioned that professional and family/friends support was important.
		Setting: Canada		
		Subjects: 2484 older women		
		Age range: 55–95 years		
Ervin [47] United States	Present the HEI scores of older adults and the association with various socio-demographic factors	Design: cross-sectional	HEI calculated from 24-h dietary recall interviews; sociodemographic and health factors obtained from household interview questionnaires	There were differences in the HEI scores depending on gender, age, ethnicity, education, smoking status and oral health.
		Setting: US		
		Subjects: 3060 older adults		
		Age range: ≥60 years		
Dean et al. [44] United Kingdom and Denmark	Investigate the influence of resources and food related goals on the food choice of older adults.	Design: cross-sectional	Questionnaire-based surveys about 11 food related goals and 22 food resources	Income, living arrangement, health status and access to a car influenced the variety of food choice of older adults. More than the actual resources, the perceived food resources also affected food choice. Food related goals also contributed to the variety of food intake.
		Setting: Europe (Poland, Portugal, United Kingdom, Germany, Sweden, Denmark, Italy, and Spain)		
		Subjects: 3200 older adults		
		Age range: ≥65 years		
Ervin and Dye [54] United States	Examine the relationship between functional dentition and HEI scores and nutrient intake in older adults.	Design: cross-sectional	HEI calculated from 24-h dietary recall interviews; sociodemographic and health factors obtained from household interview questionnaires; data about dentate status obtained from oral health examinations	Elderly men with a functional dentition consumed somewhat higher amounts of fruit and had a higher intake of alpha and beta-carotene, females had higher intakes of vitamin C.
		Setting: US		
		Subjects: 2560 older adults		
		Age range: ≥60 years		
Hunter and Worsley [45] Australia	Explore if various groups including older adults will know how to manage and maintain a healthy diet in case of lower incomes after retirement.	Design: Cross-sectional	Data about food habits at the present and future obtained from a survey; socio-demographic data	Older adults expected to maintain their practices and not changing their eating behaviors in the future.
		Setting: Australia (Melbourne)		
		Subjects: 126 older adults		
		Age range: 60–80 years (younger groups were included and compared)		
Savoca et al. [46] United States	Characterize the diet of an ethnically diverse population-based sample of older adults.	Design: cross-sectional	HEI calculated from dietary intake data from FFQ completed during home visits	African Americans had higher HEI scores compared to non-Hispanics and American Indians. Different ethnic groups significantly consumed specific food groups in different amounts. Overall diet quality was low.
		Setting: US		
		Subjects: 635 older adults		
		Age range: ≥60 years		
García and Grande [22] Spain	Explore the most important determinants for food expenditure in elderly consumers.	Design: cross-sectional	Socio-demographic data obtained from the Continuous Household Budget Survey	Type of household, education level proxying for income and consumer age influenced the level of food expenditure of elderly.
		Setting: Spain		
		Subjects: 1974 older adults head of household		
		Age range: ≥65 years		
Huy et al. [64] Germany	Explore the association between perceptions of aging and health behavior and identify determinants of a healthy diet.	Design: cross-sectional	Data about attitudes on age and aging, lifestyle (dietary habits, exercise, smoking, alcohol consumption) and sociodemographic variables obtained from a questionnaire performed through computer-assisted telephone interviews	Three perceptions of age and aging were identified: “fit and performance-hungry”, “conventional”, and “easy-going and committed”. Older adults in the first category performed physical exercise more often and were more likely to follow a healthy diet. Following a healthy diet was associated with attitudes and beliefs and other healthy behaviors.
		Setting: Germany		
		Subjects: 2002 older adults		
		Age range: 50–70 years		
Lundkvist et al. [72] Sweden and United Kingdom	Explore how older adults reflect, interpret, and express their perspectives about healthy eating messages and how they describe this in relation to managing their diets and strategies adopted.	Design: qualitative	In-depth interviews about the connection between food and health and management of a healthy everyday life; socio-demographic data	Participants had their own interpretations of dietary recommendations and reported specific and often simplified ways to manage healthy eating.
		Setting: Europe (Sweden, United Kingdom, Denmark, Germany, Italy, Poland, and Spain)		
		Subjects: 564 older adults		
		Age range: 65–98 years		
Savoca et al. [55] United States	Quantify food avoidance and modification due to oral health problems and identify foods associated with these self-management behaviors. Explore the association between food practices and diet quality.	Design: cross-sectional	Demographic and HEI from FFQ collected in home visits, avoidance and food modification assessment for specific foods	Food avoidance and modification was a common practice due to oral health problems. This was associated with a lower diet quality. Participants avoiding 3–14 foods consumed more saturated fats and added sugars. Participants modifying 4–5 foods consumed less saturated and solid fat.
		Setting: US (Rural North Carolina)		
		Subjects: 635 older adults		
		Age range: ≥60 years		
Quandt et al. [56] United States	Quantify the prevalence of dry mouth, the association between dry mouth and diet quality and between dry mouth and self-reported dietary accommodations due to oral health impairments.	Design: cross-sectional	11-item Xerostomia Inventory, HEI from FFQ, data about food modification or avoidance because of oral health problems collected from a survey	Dry mouth was positively associated with female gender, having a low income and low education. Dry mouth was not associated with overall diet quality but with a lower intake of whole grains and a higher intake of fruit.
		Setting: US (Rural North Carolina)		
		Subjects: 622 older adults		
		Age range: ≥60 years		
Ervin and Dye [58] United States	Explore the relationship between number and type of teeth and nutrient intake in older adults.	Design: cross-sectional	Dietary recall interviews, sociodemographic and health factors obtained from household interview questionnaires; data about dentate status obtained from oral health examinations: four discrete dental status groups from dental examination (complete natural dentition, incomplete natural dentition, complete mixed dentition, and incomplete mixed dentition)	Various demographic factors influenced dietary intake. A threshold number of teeth influenced nutrient intake. However, the type of teeth did not have an influence on nutrient intake.
		Setting: US		
		Subjects: 4984 older adults		
		Age range: ≥60 years		
Giuli et al. [70] Italy	Analyze dietary habits and the influence of SES on these in a sample of older adults.	Design: cross-sectional	Lifestyle data obtained from lifestyle questionnaires; nutritional data obtained from FFQ	Age was negatively correlated with all food categories. Education level and economic status were correlated with the consumption of meat, fish and dairy. There were also sex differences, men had higher consumptions of various foods including fish, meat, and fruits than women.
		Setting: Italy (Marche region)		
		Subjects: 306 older adults		
		Age range: ≥65 years		
Provencher et al. [95] Canada	Compare home and clinic performances on meal preparation tasks in a group of frail elderly.	Design: cross-sectional	Performance assessment of self-care skills; data about demographic, physical, cognitive, psychological and environmental characteristics	Participants had higher scores at home for heating soup and cutting fruit when comparing with the performances in the clinic. Better performances at home were associated with lower education level, poorer executive functions and similarity between clinic and home settings.
		Setting: Canada		
		Subjects: 33 frail older adults		
		Age range: ≥65 years		
Best and Appleton [59] United Kingdom	Explore the factors associated with the consumption of high-protein foods among older adults.	Design: qualitative	Focus group discussions about reasons for the consumption of high-protein foods	Many of the reasons for the reduced consumption of high-protein foods include chemosensory changes, dental and other physical disabilities, and changes in living situations.
		Setting: United Kingdom		
		Subjects: 28 older adults		
		Age range: ≥65 years		
Brownie and Coutts [97] Australia	Explore elderly perceptions and practices about healthy diets.	Design: qualitative	Focus group discussions about elderly perceptions and practices related to eating healthy in old age	Four main themes were found in the focus group discussions: “healthy foods”, “quantity”, “personal circumstances”, and “good intention”. Participants were not knowledgeable about the nutrient intake recommendations for older adults.
		Setting: Australia (Northern NSW)		
		Subjects: 29 older adults		
		Age range: 60–93 years		
Johnson [94] United States	Review evidence base for factors that influence diet in old age (food insecurity, nutrient intake and obesity)	Design: review	Articles about factors influencing diet in older adults, intake of various nutrients and obesity	Formal and informal social networks have an influence on the diets of older adults. Community factors include accessibility to organizations, institutions, and other networks. Type of neighborhood, living in rural or urban areas and distance to food stores and restaurants are also important influences on diets of the elderly. On the policy level it is important to achieve coordination of services. Experts from different backgrounds should work together. The development of foods adapted to the elderly should be consumer led.
		Setting:		
		Subjects: Age range:		
Mõttus et al. [73] United Kingdom	Explore the association between Five-factor model personality traits, BMI and dietary patterns.	Design: cross-sectional	Dietary patterns assessed from FFQ; personality traits measured with the NEO Five-Factor inventory	Personality traits were associated with following specific dietary patterns. For example, “openness” and “extraversion” were associated with following a Mediterranean dietary pattern.
		Setting: United Kingdom		
		Subjects: 1091 older adults		
		Age range: 70 years		
Munoz-Plaza et al. [63] United States	Find factors related to food shopping among older adults from urban areas.	Design: qualitative	In-depth interviews exploring participants’ shopping, cooking, and eating habits, qualitative observations of participants’ home and neighborhood and shopping trip observations	Intrapersonal factors that influence older adults’ food choice include concerns about physical and mental health, attitudes and perceptions about available food and income. Important social determinants are help in getting and preparing meals and social contact during meals. Environmental factors that influence food choice include transport to food shops and food availability.
		Setting: US (Brooklyn, New York)		
		Subjects: 30 older adults		
		Age range: 60–88 years		
Nicklett and Kadell [61] United States	Describe determinants of food consumption and various health outcomes in older adults. Describe patterns of consumption of fruit and vegetables and variations within and between populations	Design: review	Articles about health outcomes related to fruit and vegetables intake, trends in the intake of fruit and vegetables and predictors of intake in older adults	There were a few main factors identified as predictors of fruit and vegetables consumption: health status, physical environment, gender, marital status, household composition, social support, ethnicity, SES, and nutrition knowledge.
		Setting:		
		Subjects:		
		Age range:		
Shatenstein et al. [60] Canada	Identify individual and collective attributes influencing global diet quality.	Design: cross-sectional	Dietary intake data from 3 non-consecutive 24-h diet recalls and data from questionnaires about sociodemographic characteristics, affective, and cognitive data, health conditions, perceived physical health and functional status, dietary habits, dietary attributes and community resources	Higher education, diet knowledge, number of daily meals and perceived physical health were positively correlated with diet quality. There were some sex differences, for example in women, hunger was also a significant determinant.
		Setting: Canada		
		Subjects: 1793 older adults		
		Age range: 67–84 years		
Barrington et al. [76] United States	Investigate the association between self-reported stress and nutrient intakes and dietary behaviors.	Design: cross-sectional	Data about perceived stress, dietary behaviors, biological sex, obesity status and stress vulnerability from questionnaires and dietary intake data from semi-quantitative FFQ	Higher perceived stress was associated with a higher intake of energy from fat, including high fat snacks and fast food and lower intake of energy from total carbohydrates. Perceived stress was associated with fewer eating occasions in participants with a higher perceived stress vulnerability.
		Setting: US (Western Washington)		
		Subjects: 65,235 older adults		
		Age range: 50–76 years		
Conklin et al. [69] United Kingdom	Examine the relationship between SES and financial hardship and the variety and quantity of fruits and vegetables consumed among older adults.	Design: cross-sectional	Self-reported socio-economic factors, financial hardship and other factors (self-rated general health status, smoking status, marital status, total energy intake, BMI, physical activity level, educational attainment, social class, and self-reported accommodation), fruit and vegetable variety and quantity summarized from FFQ	The variety of fruit and vegetables differed across three levels of SES indicators and three types of financial hardship. Considering SES indicators, differences in fruit and vegetable variety were larger in men. Regarding financial hardship, there were bigger differences among women for fruit variety and in men for vegetable variety. Associations with quantity were less consistent.
		Setting: United Kingdom		
		Subjects: 9580 older adults		
		Age range: 50–79 years		
Dijkstra et al. [65] Netherlands	Identify older adults’ motivations to eat healthy and explore if these are specific per sub-groups according to socio-demographic factors and demographic, lifestyle and health characteristics.	Design: cross-sectional	Motivations to Eat Healthy and participants characteristics obtained from a self-reported questionnaire	The most mentioned motivations to eat healthy included “feeling fit”, “current health” and “body weight”. Older adults with reported chronic diseases and poor self-reported health were more likely to mention “current disease” as a motivation to eat healthy. Participants from lower SES were less likely to report “to prevent diseases” and obese elderly were less likely to mention “current health” as motivations to eat healthy.
		Setting: Netherlands		
		Subjects: 1050 older adults		
		Age range: 65–80 years		
Dijkstra et al. [71] Netherlands	Describe self-reported adherence to fish, fruit and vegetables guidelines among Dutch older adults and explore the influence of SES on these.	Design: cross-sectional	Fish, fruit, and vegetables intake measured from a short FFQ, self-reported SES data: education level, household income and occupational prestige	Participants in the lowest education group adhered less often to the vegetables guideline compared to the ones in the highest education group; respondents in the lowest income group adhered less often to the fish and fruit guidelines compared to the highest income group.
		Setting: Netherlands		
		Subjects: 1057 older adults		
		Age range: 55–85 years		
Ford et al. [57] United States	Explore the association between diet related practices and BMI with diet quality in older adults from rural areas.	Design: cross-sectional	Diet quality measured with a Dietary Screening Tool, self-reported diet related practices	Individuals with a higher diet quality were more likely to be food sufficient, eat breakfast, not have chewing difficulties and not have a decline in food intake over the past 6 months. Lower diet quality was associated with a lower BMI and poor diet-related practices.
		Setting: US (rural areas in Pennsylvania)		
		Subjects: 4009 older adults		
		Age range: ≥74 years		
Harrington et al. [68] Ireland	Identify subgroups of elderly with similar dietary patterns, explore changes in dietary patterns over a period of 10 years and the relationship with sociodemographic factors and health outcomes.	Design: prospective cohort	Anthropometric and other physical measurements, health and lifestyle data from a questionnaire, dietary intake data from FFQ at baseline and after 10 years	Three dietary patterns were identified: “Healthy”, “Western” and “Low-energy”. Most elderly remained stable in their dietary patterns over the 10 years, yet dietary pattern changes were mostly towards the “Healthy” one. Higher education was related with a change to the “Healthy” dietary pattern and lower education with remaining in an unhealthy dietary pattern.
		Setting: Republic of Ireland		
		Subjects: 923 older adults at baseline and 320 after 10-year follow-up		
		Age range: 50–69 years at baseline		
Mobley et al. [62] United States	Determine attitudes, beliefs, and barriers for adequate milk consumption in elderly women with a low income.	Design: qualitative study	Focus groups grounded in the Social Cognitive Theory exploring personal, behavioral, and environmental factors related to milk consumption	Themes included a positive attitude for the taste of milk and a primary belief that it was healthy. A barrier mentioned were gastrointestinal effects.
		Setting: US (metropolitan area)		
		Subjects: 59 older women		
		Age range: ≥60 years		
Somers et al. [75] Australia	Explore food involvement and its influences among older adults.	Design: cross-sectional	Bell and Marshall’s Food Involvement Scale, questions about socio-demographic, social and hedonic factors	Food mavenism and pleasure motivation were stronger than demographic factors in predicting food involvement.
		Setting: Australia		
		Subjects: 1041 older adults		
		Age range: ≥55 years		
Tyrovolas et al. [67] Greece	Evaluate determinants of successful aging and dietary habits in relation with healthcare facilities use among elderly living in a Mediterranean area.	Design: cross-sectional	Successful aging index (from 0 to 10) including various components: number of risk factors for cardiovascular disease, education, financial status, physical activity, BMI, self-reported Geriatric Depression Scale, frequency of social activities and yearly excursions and adherence to the Mediterranean diet	There were three main components of the aging index for successful aging: psychosocial-economic, bioclinical and lifestyle. Sex related differences, place of residence (urban or rural) and other socio-cultural determinants may impact healthy eating behaviors.
		Setting: 21 Mediterranean islands and rural Mani region		
		Subjects: 2663 older adults		
		Age range: 65–100 years		
Vella et al. [74] Canada	Explore the need for information about functional foods among older adults and awareness and perceptions about the claims in the labels of functional foods.	Design: cross-sectional	Data about consumption of functional foods, motivations for consumption, perceived information needs, information sources and awareness about health claims obtained from a questionnaire	Consumption of functional foods was high among older adults and knowledge and awareness were referred as factors that could promote the consumption of these foods. More than half of the participants would like to have more information about functional foods and most participants were aware of health claims, particularly the ones with a higher education.
		Setting: Canada		
		Subjects: 200 older adults		
		Age range: ≥60 years		
Irz et al. [66] Finland, Sweden, United Kingdom, and Italy	Identify socio-economic determinants of diet quality among older adults.	Design: cross-sectional	Diet Quality Index, socio-economic data	Diet quality was low and varied among individuals from different countries. In Finland and the United Kingdom resource availability was negatively correlated with diet quality. In the four countries, not living alone, being a female and education level were positively correlated with diet quality. There were also regional differences within countries.
		Setting: Finland, United Kingdom, Italy, and Sweden		
		Subjects: (one and two-persons households of older adults) Finland: *n* = 2994; United Kingdom: *n* = 4749; Italy: *n* = 7564: Sweden: *n* = 2023		
		Age range: ≥50 years		
Atkins et al. [92] United Kingdom	Explore childhood and adulthood socio-economic factors and social relationships as determinants of diet quality in older men.	Design: cross-sectional	Fruit and vegetable consumption data from FFQ, diet quality measured by the Elderly Dietary Index, adult and childhood socio-economic measures and social relationships	Socio-economic factors in childhood and adulthood (including manual social class, marital status and not living alone) were correlated with overall diet quality among elderly men.
		Setting: United Kingdom		
		Subjects: 4252 older men		
		Age range: 60–79 years		
Conklin et al. [87] United Kingdom	Access the associations between various combinations of economic resources, social relationships and healthy eating among older adults.	Design: cross-sectional	FFQ, six economic factors (education, social class, home ownership, money for needs, frequency of insufficient money for food/clothing and paying bills), three social relationships (marital status, living arrangement and contact with friends)	Both economic and social factors were associated with variety of consumption of fruits and vegetables. Combinations of low economic factors with lack of social relationships presented an even lower variety of consumption of fruits and vegetables than when analyzing economic and social factors separately.
		Setting: United Kingdom		
		Subjects: 9580 older adults		
		Age range: ≥50 years		
Dijkstra et al. [80] Netherlands	Identify barriers for meeting the guidelines for the consumption of fish, fruit and vegetables and identify SES differences in these barriers.	Design: cross-sectional	Level of education and household income, dietary intake measured with FFQ, barriers for dietary intake measured with self-reported lifestyle questionnaires	About half of the participants perceived barriers for the adherence to the guidelines for the consumption of fish, fruit, and vegetables. The barriers that were pointed out most often were the price of fish and fruit and lack of appetite for the consumption of vegetables. Older adults from lower SES perceived more barriers and did not meet the guidelines as much.
		Setting: Netherlands		
		Subjects: 1057 older adults		
		Age range: 55–85 years		
Granic et al. [79] United Kingdom	Characterize dietary patterns and explore the relationship between SES indicators and dietary patterns in older elderly.	Design: cross-sectional	24-h multiple pass recalls from which dietary patterns were derived, sociodemographic and lifestyle data	Three dietary patterns were identified: “High Red Meat”, “Low Meat” and “High Butter”. Older adults following the “Low Meat” dietary pattern had a higher SES, were healthier and more physically active than the older adults following the other dietary patterns. Lower education was a predictor of following “High Red Meat” and “High Butter” dietary patterns.
		Setting: United Kingdom		
		Subjects: 793 older adults		
		Age range: ≥85 years		
Kamphuis et al. [91] Netherlands	Explore differences in food motives between socio-economic groups using a discrete choice experiment.	Design: cross-sectional	Face-to-face interviews in which a discrete choice experiment was performed to explore implicit motives of food choice	Healthiness, taste, price and time of travel to grocery shop significantly influenced older adults’ food choice. Highly educated participants rated healthiness and price as more important. Participants with higher incomes rated healthiness and taste as more important.
		Setting: Netherlands		
		Subjects: 399 older adults		
		Age range: 55–72 years		
Rugel and Carpiano [86] Canada	Test if tangible and emotional social support enable the consumption of fruits and vegetables among older adults.	Design: cross-sectional	Social support survey, fruit and vegetables intake per day, frequency of eating alone and frequency of cooking own meals	Generally, emotional/informal support were associated with adequate intake of fruit and vegetables, yet among men no form of social support was associated with fruit and vegetables consumption and among women, adequate consumption of fruit and vegetables was negatively associated with tangible support and positively associated with emotional/informal support.
		Setting: Canada		
		Subjects: 14,221 older adults		
		Age range: ≥65 years		
Allès et al. [78] France and Canada	Compare nutrient patterns among two elderly populations with a common cultural background but different living environments.	Design: cross-sectional	Diet quality and nutrient patterns measured by 24-h recalls and FFQ, data about lifestyle and socioeconomic characteristics	Three nutrient patterns were identified: “Western”, “Healthy” and “Traditional”. Individuals with a higher education and a non-physical job were more likely to follow the “Healthy” dietary pattern in both countries. In the Canadian study, living alone, having a normal BMI and being an ex-smoker were also associated with following the “Healthy” dietary pattern.
		Setting: France and Canada		
		Subjects: 3308 older adults		
		Age range: 67–84 years		



Andreeva et al. [81] France	Explore dietary patterns and their sociodemographic determinants in the elderly	Design: cross-sectional Setting: France Subjects: 6686 older adults Age range: ≥65 years	Dietary patterns derived from 24-hour dietary records, socio-demographic, and health status data	Three dietary patterns were identified: “Healthy”, “Western” and “Traditional”. Education, living arrangement, being an ex/current smoker, being overweight or obese, older than 75 years, having hypertension and living in an urban area were factors that influenced which dietary pattern elderly followed.
Bardach et al. [85] United States	Understand, from older adults’ perspective, factors that may help them achieving lifestyle changes.	Design: qualitative	In-depth semi-structured interviews about motivations and plans to improve physical activity and diet behaviors after visiting the primary care center	The likelihood of physical activity and diet related changes was linked with perceptions of old age, own motivation, and perceived confidence in capability to achieve changes.
		Setting: US		
		Subjects: 104 older adults		
		Age range: 65–95 years		
Host et al. [21] Australia	Identify articles about influences of food choice among older adults.	Design: systematic review	24 articles published between 1996 and 2014 including studies conducted in settings similar with Australia	Three broad categories were identified: “changes associated with aging”, “psychosocial aspects” and “personal resources”. These factors interact between them, influencing food choice among older adults.
		Setting:		
		Subjects:		
		Age range:		
Oemichen and Smith [90] United States	Explore food choice, food access, and food security among elderly.	Design: qualitative	Focus group discussions about how seniors make food choices and access food and about perceptions about food insecurity	Five main themes were identified from the focus group discussions: “former experiences”, “financial and food security driving use of food assistance programs”, “food access strategies”, “physical changes associated with aging” and “social influences”.
		Setting: US (2 counties with high and low Supplemental Nutrition Assistance Program participation)		
		Subjects: 62 older adults		
		Age range: ≥60 years		
Shatenstein et al. [13] Canada	Examine individual and collective factors as predictors of change in Global Diet Quality.	Design: longitudinal	Dietary intake data from non-consecutive 24-h dietary recalls at baseline and 3 years follow-up and diet quality calculated with the Canadian HEI	In men, education and hunger positively predicted diet quality change over 3 years. For women, diet knowledge was a positive predictor of diet quality change over time. Diet quality in the first year and social network were negative predictors of diet change over time for both men and women.
		Setting: Canada		
		Subjects: 373 older adults		
		Age range: 68–82 years at baseline		
Byker Shanks et al. [88] United States	Explore how the rural food environment influences food choices among older adults.	Design: qualitative	Focus group discussions about factors influencing rural older adults’ food choices	Four main themes were identified from the focus group discussions: “perception of the rural community environment,” “support as a means of increasing food access,” “personal access to food sources” and “dietary factors.”
		Setting: US (rural Montana)		
		Subjects: 33 older adults		
		Age range: ≥50 years		
Hawkesworth et al. [96] United Kingdom	Investigate the association between the neighborhood food environment and consumption of fruit and vegetables among older adults.	Design: cross-sectional	Local food environment characteristics that influence food access and availability, self-reported intake of fruits and vegetables	There was evidence for an association between area income deprivation and fruit and vegetable consumption in older adults.
		Setting: United Kingdom		
		Subjects: 2007 older adults		
		Age range: 69–92 years		
Sheats et al. [98] United States	Test the OUR Voice approach to identify influences on older adults’ ability to access, choose and buy healthy food; document participants experience while navigating the food environment and facilitate a consensus and advocacy-building process about the most salient worries of residents about food choice and purchase.	Design: qualitative	Factors that facilitate or hinder access to food and food related behaviors	Some of the major barriers for healthy eating were lack of access to affordable healthy food and transportation. Participants were trained in advocacy skills and shared the findings with policy makers.
		Setting: US		
		Subjects: 23 older adults		
		Age range: ≥60 years		
Hung et al. [82] Belgium and Netherlands	Profile older adults from the European Union regarding their appetite and protein intake and identify diet related and physical activity behaviors.	Design: cross-sectional	Appetite and protein intake strata obtained from a simplified nutritional appetite questionnaire (SNAQ)	The older adults that were more likely to have a poor appetite and lower protein intake were 70 years or more, lived in the United Kingdom or Finland, with a lower education level, reported financial difficulties, had less knowledge about protein and were fussier about food.
		Setting: Netherlands, United Kingdom, Finland, Spain, and Poland		
		Subjects: 1825 older adults		
		Age range: ≥65 years		
Rempe et al. [84] Germany	Investigate if the motives from The Eating Motivation Survey are consistent in a sample of older adults and between older adults with and without functional impairments and explore what motivates eating behavior in older adults.	Design: cross-sectional Setting: Germany Subjects: 376 older adults (149 unimpaired and 227 physically impaired participants) Age range: ≥70 years	Eating motives (short version of The Eating Motivation Survey), socio-demographic factors (age and living status), anthropometry (BMI), cognitive function (Mini Mental Status Examination) and functional status (Short Physical Performance Battery)	“Liking” was the motive most often related with eating behavior. “Health” and “Natural Concerns” were also often related with eating behavior. Eating behavior was based in the intercorrelation of various motives. “Pleasure” was correlated with all eating motives.
Van den Heuvel et al. [93] United Kingdom	Explore the association between food neophobia, physical disadvantage and demographic characteristics in older adults.	Design: cross-sectional	Food neophobia scores, physical disadvantage (denture wearing, need for help with food shopping and preparation, risk of sarcopenia), sociodemographic factors	Food neophobia was positively associated with older age, living alone and lower education level.
		Setting: United Kingdom		
		Subjects: 377 older adults		
		Age range: ≥55 years		
Watson et al. [77] United Kingdom	Access how dental status influences nutrient intake, nutritional status, and perceived ability to eat specific foods in older adults.	Design: cross-sectional	Dietary intake assessed by a 4-day food diary, socio-demographic, health behavior and oral health data, blood nutrient concentrations	Older adults wearing dentures (with and without dentition) reported more difficulties eating certain foods. Older adults without dentition wearing dentures had lower intakes of certain micronutrients than the ones with dentition that did not wear dentures.
		Setting: United Kingdom		
		Subjects: 1053 older adults		
		Age range: ≥65 years		
Howell [89] United States	Explore the relationship between sociocultural factors that influence diet, physical activity and nutrition status among older adults.	Design: cross-sectional	Anthropometric measurements, dietary intake measured with Block FFQ, physical activity measured with Community Healthy. Activities Model Program for Seniors (CHAMPS) and sociocultural influences reported in a questionnaire	Generally, diet and physical activity did not meet national recommendations. Family influence seemed to increase fruit consumption and participation in sociocultural events seemed to increase the consumption of fat and sugars. Media and friends were associated with a higher energy expenditure.
		Setting: US (Alaska)		
		Subjects: 82 older adults		
		Age range: ≥65 years		
Mumme et al. [83] New Zealand	Explore the association between dietary patterns and sociodemographic and lifestyle factors in older adults.	Design: cross-sectional	Dietary patterns based on FFQ, sociodemographic and lifestyle data from questionnaires	Three dietary patterns were derived “Mediterranean”, “Western” and “Prudent”. Higher scores in the “Mediterranean” dietary pattern were associated with being female, having a higher education and being more physically active. The “Western” dietary pattern was associated with being male, having a higher intake of alcohol, not living alone, and having a secondary education. Lastly, the “Prudent” dietary pattern was associated with a higher level of physical activity and a lower intake of alcohol.
		Setting: New Zealand		
		Subjects: 367 older adults		
		Age range: 65–74 years		

Abbreviations: United States (US), Socio-economic Status (SES), United Kingdom (UK), Body Mass Index (BMI), Food Frequency Questionnaire (FFQ), Healthy Eating Index (HEI).

When analyzing the 77 publications using VosViewer, relevance scores ranged between 0.04–3.17, 0.06–4.69, 0.08–3.38, 0.05–3.75 in the publications between 2000–2004, 2005–2009, 2010–2014 and 2015–2020, respectively.

**FIGURE 3:**
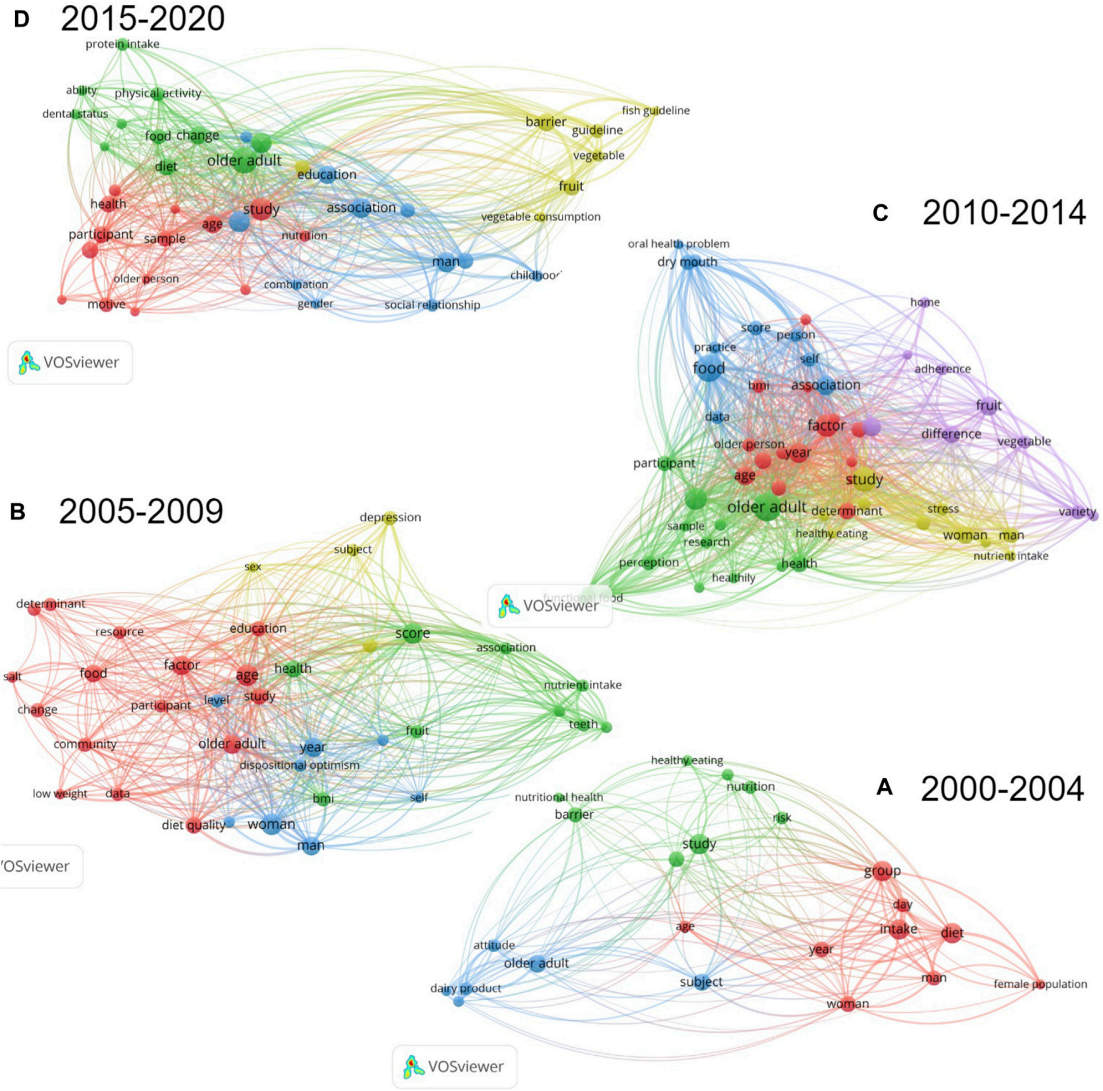
Cluster Visualization of Co-occurrence of Terms in Publications from **(A)** 2000–2004, **(B)** 2005–2009, **(C)** 2010–2014 and **(D)** 2015–2020, using Vosviewer [18] (scoping review, high-income countries, 2000–2020).

A total of 16 studies were published between 2000 and 2004. Among these, 23 terms co-occurred at least 5 times (Figure 3A). “Nutritional health” (3.17), “behavioral control” (2.45), “intention” (2.39) and “attitude” (1.93) were identified as frequent and relevant terms.

In the 14 articles published between 2005 and 2009, 37 terms co-occurred at least 5 times (Figure 3B) of which “functional dentition” (2.31) and “teeth” (1.92) were highly relevant. “Depression” (1.34) and “community” (0.79) also appeared frequently, however with a lower relevance score.

Among the 26 selected articles published between 2010 and 2014, a total of 58 terms co-occurred at least 5 times (Figure 3C). Herein, some of the most relevant terms were “information” (3.04), “oral health problem” (2.69) and “dry mouth” (2.08). “Food involvement” (0.84) also appeared although with a somewhat lower relevance score.

In the 21 studies published between 2015 and 2020, 43 terms occurred at least 5 times (Figure 3D) with “guideline” (2.81), “childhood” (2.20), “motive” (1.68), “dental status” (1.43) and “social relationship” (1.24) receiving quite high relevance scores.

The term “education” appeared first in the analysis of publications between 2005 and 2009; however, it had one of the lowest relevance scores and it kept appearing throughout all the periods with low relevance scores (0.11 or lower).

Some terms are not visible in Figure 3 as in case of lack of space the software automatically “hides” them.

## Discussion

### Research Between 2000–2004 Focused on Individual Determinants of Eating Behavior

When analyzing studies published between 2000 and 2004, it becomes apparent that publications focused mainly on individual determinants [26, 27]. Terms including “attitude”, “intention” and “behavioral control” were found to influence food choice among elderly individuals [27–30] (Figure 3A).

During this period, “nutritional health” was another term found in the bibliometric mapping. This was often explored as a result of individual characteristics as sex, (oral) health and ethnicity [28, 31–36]. Vitolins et al. [33] reported that African American men aged over 70 years consumed the lowest amounts of fruits and vegetables. Some studies published during this period also reported that education, marital status, social interaction and geographical differences were correlated with diet quality among older adults [26, 31, 37–41]. In a study from van Rossum et al. [38], lower educated individuals had a lower intake of fiber and a higher intake of saturated fat and cholesterol. “Resistance to change” was also referred to as an important determinant of food choice in elderly [27]. Studies further suggest that earlier life experiences may shape present food preferences. A study from Sindler et al. [29] investigating the effects in dietary habits of survivors of extreme life events like the Holocaust, found that this experience still shaped the eating behaviors at old age. Subjects reported having reserve foods at home and to avoid food waste [29]. Regarding geographical differences, Haveman-Nies et al. [37] reported that Southern European elderly had higher diet quality than Northern Europeans.

### Research Between 2005–2009 Focused on the Effect of Social Determinants on Eating Behavior

In the following period (2005–2009), a more holistic view of determinants of eating behavior started to evolve, considering not only the individual but their built “food environment”, particularly community-related aspects. In this period, demographics as sex, ethnicity, education, income, living arrangements and geographic location continued to be reported as important determinants of quality of diet in elderly [16, 42–47]. However, these were often correlated with other collective determinants.

“Community” was a frequent term found in the publications between 2005 and 2009. It was reported that traditional foods typical of a community represent their identity and it is a complex task to replace these with healthier versions [48]. Payette and Shatenstein [16] reported that determinants of healthy eating were not only individual but also collective. Social interaction and support seem to positively influence older adults’ quality of diet, such as the intake of fruit and vegetables [16, 26]. The support of family, friends and healthcare professionals was mentioned as very important by elderly women at nutritional risk [49]. A study based in the Health Promotion model, reported that not only self-efficacy but also interpersonal support were associated with healthy eating in elderly people [50]. Underweight older adults stated that social contact during meals was crucial to maintaining healthy dietary patterns, loneliness was often associated with feelings of depression that led to lower appetite and the consumption of less balanced meals [51].

“Depression” was another term found among the literature. Payne et al. [52] compared the nutrient intake between a depressed group of elderly and a control group, the depressed group had higher intakes of alcohol and cholesterol when comparing with the control [52]. Furthermore, being positive about the aging process and perceiving fewer barriers to follow a healthy diet seemed to positively influence the consumption of healthy foods [53]. A study performed by Giltay et al. [53] reported that “dispositional optimist” in older men increased the likelihood of the consumption of healthy foods such as fruit, vegetables and whole-grain bread [53]. It has also been suggested that having food related goals as “cooking for others” were significant predictors of a varied diet [44].

“Teeth” and “functional dentition” were also frequent terms reported in the visual mapping from VosViewer. It has been shown that having a functional dentition significantly improves the consumption of fruits and vegetables in elderly males [54].

### Research Between 2010–2014 Assessed Determinants of Food Choice as Life Experiences, Information and “Food Involvement”

Among publications between 2010 and 2014, “oral health problem” and “dry mouth” were oral health related terms that kept on appearing at the bibliometric mapping, these lead to problems as chewing difficulty and xerostomia [55–57]. It is likely that denture wearers and/or elderly with a low number of teeth either avoid foods that would create discomfort or use food modification techniques [55, 56, 58]. Quandt et al. [56] found a significant correlation between suffering from dry mouth and having a lower consumption of whole-grain foods. It has been proposed that taste and smell impairments that accompany the process of aging are related to sensory perception of food [59]. This in turn has been suggested to affect appetite [60, 61].

There were other recurring topics in the publications between 2010 and 2014. Attitudes and beliefs towards food were, once again, shown to influence eating behavior [62–64]. Individual motivations to eating healthy were described by Dijkstra et al. [65]. Elderly reported “Health”, “Feeling fit” and “Body weight” as the most important determinants of food choice [65]. Sex, socio-economic status, living arrangements, education, ethnicity and place of residence were also described as influencing eating behavior in elderly [59–61, 65–71]. It was reported that older adults with lower education level and income adhered less to vegetables and fish/fruit government guidelines respectively [71].

“Information” was another term that appeared between 2010 and 2014. In a study performed with elderly from several European countries it was reported that even though older adults are aware of information related to nutrition recommendations and open to follow them, they draw their own conclusions from these and try to make it fit their lifestyle, habits and culture [72]. Traditional and familiar foods, often resulting from family habits and other life-course experiences, have been shown to be the preferred by the majority of older adults [72]. Several studies reported that dietary patterns of older adults are bound to their cultural and ethnic background [61]. For instance, older adults living in Mediterranean countries typically have a higher consumption of fruits and vegetables when comparing with older adults from the United States [61]. Studies further suggest that open and curious elderly are more likely to adopt novel dietary habits [73]. Older adults report that advice and provision of information from healthcare professionals have the potential to highly influence their (functional) food consumption [74]. It seems that there is an opportunity for healthcare professionals to provide nutrition information to older adults with the potential to modulate their food choices, yet this needs to be tailored to the characteristics of each target group [74]. Furthermore, it is important to understand the process of interpretation of nutrition information of different sub-groups of elderly to communicate with them in a successful way [72].

The concept denominated “Food involvement” was another term reported in the bibliometric mapping. Sommers et al. [75] investigated this concept that is characterized as “the level of importance of food in a person’s life” [75]. Pleasure was found to be positively correlated with “food involvement” [75]. Additionally, studies suggest that higher perceived stress is positively related with unhealthy eating habits such as high intakes of saturated fats and sugars, further highlighting the importance to emphasize pleasurable aspects of eating to motivate older adults to follow healthy diets [75, 76].

### Research Between 2015–2020 Assessed Social Determinants, the Effect of Previous Experiences on Food Choice and Barriers to Follow Government Nutritional Guidelines

When exploring the last cluster of publications, “dental status” was once again a frequent term among literature. There was a focus on micronutrient intake that was significantly lower among denture wearers without any natural dentition [77]. “Education” and other socio-demographic factors were, once more, recurring themes. Several studies, suggest that commonly, highly educated older adults tend to follow or adopt healthier dietary patterns [13, 78–83].

“Motive” was another term frequently present in the most recent literature. In a study performed by Rempe et al. [84], the so called “Eating Motivation Survey” was applied to determine these motivations in older adults [84]. According to the results of this study, “Health”, “Natural concerns” and “Weight control” are among the greatest food choice influences [84]. In a study by Bardach et al. [85], older adults were asked which factors would be important for them to change their nutrition and physical activity behavior. The study revealed three main individual factors: “perceptions of old age, personal motivation and perceived confidence in ability to make effective changes” [85]. It has been discussed that it is likely that older adults with health impairments that experienced the benefits of improving health behaviors are more motivated to follow a healthy diet than the ones that do not feel that an improvement is possible [85]. Employing the ‘Extended Parallel Process Model’ it was pointed out that if individuals do not believe that they can respond to a threat (e.g., health decline due to older age) they will come to terms with it instead of trying to aim for a change [85].

“Social relationship” was also among the frequent terms in the literature mapping. Rugel and Carpiano [86] hypothesize that emotional and informational support may enhance women’s motivation to eat in a healthy way by increasing their perception of self-efficacy and sense of purpose. A lack of social relationships, particularly when associated with low economic resources appeared to influence eating behavior in older adults, especially men, in a study performed in the United Kingdom [87]. A group of older adults living in a rural area reported “community support” to be of major importance in their food choices [88]. In fact, family influence was found to positively impact the consumption of fruit and vegetables among a group of older adults from the United States [89]. Participants were familiar with food sharing and trading with friends and family; peers were often relied upon as source of assistance [88, 90].

“Guideline” was another term appearing in the bibliometric mapping of publications between 2015 and 2020. It seems that participants from lower socio-economic status perceive more barriers to follow nutritional guidelines [80]. The high price of healthy foods is often referred to as a barrier for their consumption [80, 82]. Besides, sensory appeal is mentioned in literature as important for food choice [91]. It seems that disliking healthy foods such as fruits and vegetables constitutes a barrier for their consumption by older adults, particularly, the ones with lower incomes [80, 91].

“Childhood” was another frequent term found in literature published in this period. Studies suggest that in older men, an important factor that seems to influence food choice are childhood experiences [92]. This agrees with the findings reported above regarding the influence of life-course experiences on the eating behavior of the elderly. Traditional and familiar foods, often resulting from family habits and other life-course experiences, have been shown to be the preferred foods for the majority of older adults [88, 90]. Despite this, in recent years, the food industry has started the development of novel foods adapted to the needs of older adults e.g., being of softer consistencies and fortified; however, these kind of products may not be well accepted due to the stigmatization and even difficulty accepting new foods—food neophobia [93].

### After 2010 Some Studies Investigated (Physical) Barriers for Healthy Eating

A physical determinant that affects the characteristics of food environments and food choices of older adults is the difference between rural and urban settings [94]. Epidemiological studies suggest that in rural settings older adults often suffer from limited infrastructures which hinders their access to healthy food [59, 61]. Studies also suggest that transportation reliability is an important factor for accessing food/food related programs [61, 63]. It is frequently mentioned by the elderly that cheaper food shops are not accessible to them, particularly for the ones that have impaired mobility [59, 61]. The characteristics of the place of residence, including the type of foods available, cost and proximity or availability of transport to food shops, seem to influence the food consumption of older adults [59, 63, 67, 94]. Furthermore, the environment where older adults prepare and eat their meals is also of relevance [95]. A study performed by Provencher et al. [95], reported that the elderly scored better at heating soup and cutting fruit at their own homes when compared to a kitchen in a clinic. Regarding community initiatives such as meal provision and government assistance, it was reported that low participation may result from lack of knowledge and stigma around these initiatives [88, 90, 94]. The fact that meals may not be tasteful or culturally sensitive has also been reported to lead to a lower utilization of these services [63].

### Suggestions for Nutrition Communication Strategies

Several studies suggest that communication strategies need to be culturally tailored [82, 89]. A study performed by Howell [89] reported that the socio-cultural environment has a high influence on personal beliefs and attitudes towards diet. Moreover, studies suggest that the socio-cultural environment may widely vary between countries [46, 82, 89]. However, different neighborhoods may also need adapted communication strategies. A cross-sectional study from the United Kingdom investigated the influence on food choice of measures as food retail diversity, transport provision, marketing of unhealthy food and physical infrastructures that may affect mobility for older people [96]. In this study it was reported that “area (income) deprivation” is associated with the intake of fruit and vegetables among elderly [96].

Studies suggest that nutrition messages should be clear, short and targeted to the needs of specific target groups [59]. It further has been suggested that public policy interventions meant to improve the food choices of older adults should not only communicate the importance of maintaining a healthy diet but be actionable, explaining which actions can be taken by the elderly [97]. “Standalone” communication about healthy eating have been reported to be less effective than desired, but rather communication employing practical actions (e.g., nutrition workshops) may be more effective [59, 82]. Furthermore, it has been shown that interventions should focus on targeting older adults’ distrust of information sources such as food labels and media channels [74]. Additionally, it has been proposed that it is important to include the elderly in nutrition policy discussions in a citizen science approach, allowing them to share their perspectives [98].

### Translation to Practical Communication Strategies

• Adapt for sex differences as older women and men typically eat differently and may even have differences in socioeconomic status.
• Consider hedonic characteristics of food simultaneously with practical matters as cost.
• Recognize oral health and other physiological impairments common at old age.
• Do not only communicate the importance of maintaining a healthy diet but explain which actions can be taken to achieve this.
• Create culturally tailored strategies, adapted by country and culture.
• Explore opportunities and barriers for using different communication channels, including digital channels.
• Use citizen science approaches in which older adults are included in the creation of communication strategies.
• Be dynamic and sensitive for changes in the food environment, it is essential to understand the past to target eating behavior in the present.

### Conclusion and Future Research

Research about ecological determinants of eating behavior in older adults has evolved over time, leading to a holistic view of behavior including all ecological levels. As summarized in this review, studies have reported that within this age group there are differences regarding culture, living environment and socio-demographic characteristics. These have been proposed to shape older adults’ food choice. While various theories have been developed to explain eating behavior for different age groups, for elderly, tailored measures to communicate healthy nutrition are still limited. It is important to explore the perspective of elderly towards their own behaviors and consider how to target external barriers for healthy eating.

We live in a constantly changing society; hence it is important to understand how to use the available communication resources to improve the health of this growing segment in society.

Research about eating behavior in the elderly, and ecological determinants that influence it, is crucial to achieve health and quality of life in this group. As the food environment evolve throughout the years, future research should stay updated about how various periods and events affect older adults. This will help to gain insights into the best strategies to communicate about nutrition with older adults with diverse backgrounds and modulate their eating behaviors towards healthier ones if needed.

### Limitations

As data about the effects of the pandemic on the eating behavior of the elderly is still limited, this review only focused on the period before that. Furthermore, only studies performed in community-dwelling older adults were included in this review. This choice was made as targeting institutionalized elderly would require investigating different stakeholders and determinants. The age range of the elderly in the studies included in this review is quite variable, yet it was decided to consider each study’s definition of older adult, as the objective of this scoping review was to gather information as broad as possible. Lastly, although an internal review protocol was elaborated, this was not registered in any open platform.

### Strengths

It is challenging to deliver an understandable “picture” of the determinants of eating behavior in the elderly. Here, both *a-priori* and *a-posteriori* analyses were performed with the support of graphical representations to explore determinants of eating behavior in a holistic way, thereby creating a basis for the development of nutrition communication strategies focusing on this group. Based on an in-depth analysis of the literature, this review provides advice for the development of nutrition communication strategies for the elderly. It is important to acknowledge that the topic of this review is dynamic, hence it is necessary to capture past influences in eating behavior to understand the present and make future predictions. This was done by splitting the literature into periods and analyzing patterns emerging in those.
