# Supplementary material for: Beyond the Individual -A Scoping Review and Bibliometric Mapping of Ecological Determinants of Eating Behavior in Older Adults
Source: Public Health Rev. 2022 Aug 3;43:1604967. doi: 10.3389/phrs.2022.1604967 (PMC9381692; doi:10.3389/phrs.2022.1604967)
Supplement: Supplementary file 1 [file Datasheet1.docx]

**Supplementary material - Ecological determinants of eating behavior in older adults grouped in categories, per period**

**Year Cluster 2000-2004**

| **Publications** | **Most prominent determinants mentioned** | **Topic** |
| --- | --- | --- |
| **Individual level** | | |
| Kim et al. (2003) | attitudes | Cognitive perceptions  n=4 |
| De Almeida et al. (2001) | resistance to change |  |
| De Almeida et al. (2001) | self-control |  |
| Kim et al. (2003) | perceived behavioral control |  |
| McDonald et al. (2000) | self-care capacity | Behavioral factors/skills  n=3 |
| McDonald et al. (2000) | being able to drive |  |
| Tucker and Reicks (2002) | exercise as gateway behavior |  |
| Haveman-Nies et al. (2001) | general lifestyle practices | Lifestyle practices  n=2 |
| Pryer et al. (2001)  Rurik (2004) | being a (non/ex) smoker |  |
| Vitolins et al. (2002)  Palmer (2003)  Österberg et al. (2002) | oral health | Health status  n=2 |
| Callen and Wells (2003)  Rurik (2004) | health problems |  |
| McDonald et al. (2000) | financial security | Socio-demographic characteristics  n=6 |
| Johnson and Garcia (2003) | immigrant status |  |
| Rurik (2004)  Pryer et al. (2001) | gender |  |
| Vitolins et al. (2002) | ethnicity |  |
| Van Rossum et al. (2000)  Rurik (2004) | education level |  |
| McDonald et al. (2000)  Rurik (2004) | marital status |  |
| Sindler et al. (2004) | past traumatic events | Previous life experiences  n=1 |
| **Social level** | | |
| McDonald et al. (2000) | informal social networks | Inner social networks support  n=2 |
| Rurik (2004) | housing situation |  |
| McDonald et al. (2000)  Rurik (2004) | being socially active | General social support  n=5 |
| McDonald et al. (2000) | formal social networks |  |
| Pryer et al. (2001) | social profile |  |
| De Castro (2002) | number of other people present at meals |  |
| Callen and Wells (2003) | social connectedness |  |
| **Physical level** | | |
| Haveman-Nies et al. (2001) | geographic differences | Geographical environment influence  n=4 |
| Corrêa Leite et al. (2003) | area of residence |  |
| McDonald et al. (2000) | rural environment |  |
| Vitolins et al. (2002) | food gardens |  |
| McDonald et al. (2000) | local restaurants as food sources | Food sources, access and availability  n=3 |
| De Almeida et al. (2001) | quality of food (e.g., freshness) |  |
| De Castro (2002) | location of meals |  |
| **Macro level** | | |
| De Almeida et al. (2001) | cost of food | Food policy  n=2 |
| Corrêa Leite et al. (2003) | educational interventions targeted at general healthy behavior |  |
| De Castro (2002) | palatability of meals delivered | Food assistance programs  n=1 |

**Year Cluster 2005-2009**

| **Publications** | **Most prominent determinants mentioned** | **Topic** |
| --- | --- | --- |
| **Individual level** | | |
| Walker et al. (2006) | self-efficacy | Cognitive perceptions  n= 8 |
| Giltaya et al. (2007) | dispositional optimism |  |
| Payette and Shatenstein (2005) | health awareness |  |
| Payette and Shatenstein (2005) | knowledge |  |
| Payette and Shatenstein (2005) | attitudes |  |
| Payette and Shatenstein (2005) | beliefs |  |
| Dean et al. (2009) | food knowledge |  |
| Dean et al. (2009) | perception of the distance to the shops |  |
| Payette and Shatenstein (2005) | behaviors | Behavioral factors/skills  n=1 |
| Shannon et al. (2007) | using dietary supplements | Lifestyle practices  n=2 |
| Shannon et al. (2007) | smoking |  |
| Payne et al. (2006) | depression | Health status  n=11 |
| Ervin and Dye (2009) | having no teeth/wearing dentures |  |
| Payette and Shatenstein (2005) | vision |  |
| Payette and Shatenstein (2005) | audition |  |
| Payette and Shatenstein (2005) | olfaction |  |
| Payette and Shatenstein (2005) | decline in salivary flow |  |
| Payette and Shatenstein (2005) | masticatory impairment |  |
| Shannon et al. (2007) | body mass index |  |
| Ervin (2008) | oral health |  |
| Dean et al. (2009) | health status |  |
| Dean et al. (2009) | level of appetite |  |
| Payette and Shatenstein (2005)  Shannon et al. (2007)  Ervin (2008) | age | Socio-demographic characteristics  n=7 |
| Rurik (2006)  Ervin (2008)  Payette and Shatenstein (2005) | gender/sex |  |
| Payette and Shatenstein (2005)  Dean et al. (2009) | income |  |
| Hunter and Worsley (2009) | changes due to lower standard of living |  |
| Payette and Shatenstein (2005)  Shannon et al. (2007)  Ervin (2008) | education |  |
| Payette and Shatenstein (2005) | widowhood |  |
| Shannon et al. (2007)  Ervin (2008)  Savoca et al. (2009) | ethnicity |  |
| **Social level** | | |
| Tannenbaum and Shatenstein (2007) | encouragement from family and friends | Inner social networks support  n=3 |
| Dean et al. (2009) | support from friends and neighbors |  |
| Martin et al. (2005) | eating alone |  |
| Dean et al. (2009) | living arrangements | Living arrangements (household)  n=1 |
| Martin et al. (2005) | social isolation | General social support  n=6 |
| Payette and Shatenstein (2005) | loneliness |  |
| Payette and Shatenstein (2005) | social status |  |
| Payette and Shatenstein (2005) | adequate social support |  |
| Walker et al. (2006) | interpersonal support |  |
| Tannenbaum and Shatenstein (2007) | encouragement from health professionals |  |
| **Physical level** | | |
| Payette and Shatenstein (2005)  Savoca et al. (2009) | rural versus urban environment | Geographical environment influence  n=3 |
| Payette and Shatenstein (2005) | local food environment |  |
| Shannon et al. (2007) | community of residence |  |
| Payette and Shatenstein (2005) | appropriate food shopping environment | Food sources, access and availability  n=5 |
| Dean et al. (2009) | access to a car |  |
| Dean et al. (2009) | access to high-quality products |  |
| Dean et al. (2009) | access to good service providers |  |
| Tannenbaum and Shatenstein (2007) | availability of low-cost food |  |
| Dean et al. (2009) | having better kitchen facilities | Others (physical level)  n=1 |
| **Macro level** | | |
| Smith et al. (2006) | cultural complexity towards food | Socio-cultural complexity  n=2 |
| Payette and Shatenstein (2005) | culture |  |
| Payette and Shatenstein (2005) | accessible food labels | Food and beverage industry  n=1 |
| Payette and Shatenstein (2005) | marketing of the "healthy eating" message | Food marketing  n=1 |
| Hunter and Worsley (2009) | relying on government pensions or allowance for financial support | Food assistance programs  n=2 |
| Payette and Shatenstein (2005) | provision of effective, community-based meal delivery services |  |

**Year Cluster 2010-2014**

| **Publications** | **Most prominent determinants mentioned** | **Topic** |
| --- | --- | --- |
| **Individual level** | | |
| Huy et al. (2010) | perceptions of aging | Cognitive perceptions  n=15 |
| Provenchera et al. (2012) | cognitive functions |  |
| Brownie and Coutts (2013) | perceived healthfulness of foods |  |
| Mõttus et al. (2013) | personality traits (openness, extraversion, neuroticism, agreeableness, and conscientiousness) |  |
| Shatenstein et al. (2013) | perceived physical health |  |
| Barrington et al. (2014) | stress |  |
| Dijkstra et al. (2014) | motivations to eat healthily (“feeling fit”, "current health" and "body weight") |  |
| Mobley et al. (2014) | attitude |  |
| Somers et al. (2014) | food mavenism |  |
| Vella et al. (2014) | awareness/knowledge about functional foods |  |
| Lundkvist et al. (2010) | own concept of healthy eating |  |
| Munoz-Plaza et al. (2013) | attitudes and perception about food availability |  |
| Mobley et al. (2014) | belief and barriers about milk consumption |  |
| Somers et al. (2014) | pleasure motivation |  |
| Somers et al. (2014) | food involvement |  |
| Dijkstra et al. (2014) | adherence to the fruit, vegetables and fish guidelines | Behavioral factors/skills  n=4 |
| Nicklett and Kadell (2013)  Shatenstein et al. (2013) | dietary knowledge |  |
| Ford et al. (2014) | skipping breakfast |  |
| Lundkvist et al. (2010) | healthy eating management in daily life |  |
| Shatenstein et al. (2013) | alcohol consumption | Lifestyle practices  n=1 |
| Savoca et al. (2010) | food avoidance and modification due to oral health problems | Health status  n=15 |
| Best and Appleton (2013) | restricted mobility and disabilities affecting food preparation and cooking |  |
| Quandt et al. (2011) | dry mouth |  |
| Ervin and Dye (2012) | number of natural and prosthetic teeth |  |
| Best and Appleton (2013) | chemosensory function |  |
| Munoz-Plaza et al. (2013)  Nicklett and Kadell (2013) | physical and mental health |  |
| Best and Appleton (2013) | dental abilities |  |
| Brownie and Coutts (2013) | health conditions |  |
| Johnson (2013) | polypharmacy |  |
| Munoz-Plaza et al. (2013) | ability to walk |  |
| Shatenstein et al. (2013) | wearing dentures |  |
| Shatenstein et al. (2013) | hunger/appetite |  |
| Dijkstra et al. (2014) | obesity |  |
| Ford et al. (2014) | decline in food intake |  |
| Ford et al. (2014) | chewing difficulties |  |
| Giuli et al. (2012) | economic status | Socio-demographic characteristics  n=10 |
| Conklin et al. (2014) | financial barriers |  |
| Ford et al. (2014) | being food insecure |  |
| Garcia and Grande (2010)  Giuli et al. (2012)  Shatenstein et al. (2013)  Dijkstra et al. (2014)  Irz et al. (2014) | education |  |
| Garcia and Grande (2010)  Giuli et al. (2012) | age |  |
| Giuli et al. (2012)  Nicklett and Kadell (2013)  Irz et al. (2014) | gender |  |
| Munoz-Plaza et al. (2013)  Dijkstra et al. (2014) | income |  |
| Nicklett and Kadell (2013) | marital status |  |
| Nicklett and Kadell (2013) | race/ethnicity |  |
| Nicklett and Kadell (2013)  Dijkstra et al. (2014) | socioeconomic status |  |
| Best and Appleton (2013) | previous experiences in life | Previous life experiences  n=1 |
| Best and Appleton (2013) | need to cook | Others (individual level)  n=1 |
| **Social level** | | |
| Johnson (2013) | education of individuals and caregivers about available resources through service providers in caregiving roles | Inner social networks support  n=1 |
| Garcia and Grande (2010) | type of household | Living arrangements (household)  n=4 |
| Irz et al. (2014) | not living alone |  |
| Best and Appleton (2013) | changes in living situation |  |
| Nicklett and Kadell (2013) | household composition |  |
| Johnson (2013) | formal and informal social networks | General social support  n=5 |
| Brownie and Coutts (2013) | social situations |  |
| Munoz-Plaza et al. (2013) | help in obtaining and preparing meals |  |
| Munoz-Plaza et al. (2013) | social contact during meals |  |
| Nicklett and Kadell (2013) | social support |  |
| **Physical level** | | |
| Tyrovolas et al. (2014) | place of residence | Geographical environment influence  n=6 |
| Provenchera et al. (2012) | familiar environment |  |
| Johnson (2013) | living in rural areas |  |
| Johnson (2013) | living in safe neighborhoods |  |
| Nicklett and Kadell (2013) | geographic/ physical environment |  |
| Irz et al. (2014) | regional differences |  |
| Best and Appleton (2013) | restricted mobility including access to shops | Food sources, access and availability  n=6 |
| Best and Appleton (2013) | suitable bus routes to improve accessibility |  |
| Best and Appleton (2013) | distance to food stores and restaurants |  |
| Johnson (2013) | types of foods available |  |
| Munoz-Plaza et al. (2013) | public transportation |  |
| Munoz-Plaza et al. (2013) | restaurants as food sources |  |
| Shatenstein et al. (2013) | perishability of foods | Others (physical level)  n=3 |
| Johnson (2013) | calm eating environment |  |
| Munoz-Plaza et al. (2013) | food quality |  |
| **Macro level** | | |
| Conklin et al. (2014) | social inequalities | Socio-cultural complexity  n=2 |
| Tyrovolas et al. (2014) | socio-cultural factors |  |
| Best and Appleton (2013) | special offers on individual items | Food and beverage industry  n=2 |
| Johnson (2013) | food and supplement industry |  |
| Johnson (2013) | placing foods within visual range | Food marketing  n=1 |
| Best and Appleton (2013) | food crises | Food policy  n=3 |
| Johnson (2013) | coordination of nutrition services at policy level |  |
| Munoz-Plaza et al. (2013) | cost of foods |  |
| Johnson (2013) | cooking classes and meal clubs to encourage interaction and skills development | Food assistance programs  n=2 |
| Best and Appleton (2013) | foods provided by the government not being culturally sensitive or tasteful |  |
| Munoz-Plaza et al. (2013) | healthcare | Healthcare support  n=1 |
| Harrington et al. (2014) | longitudinal stability/transitions in dietary patterns over the years | Others (macro level)  n=3 |
| Best and Appleton (2013) | health information |  |
| Vella et al. (2014) | information about functional foods |  |

**Year Cluster 2015-2020**

| **Publications** | **Most prominent determinants mentioned** | **Topic** |
| --- | --- | --- |
| **Individual level** | | |
| Bardach et al. (2016) | perceptions of old age | Cognitive perceptions  n=9 |
| Shanks et al. (2017) | perception of the rural community environment |  |
| Van den Heuvel et al. (2019) | food neophobia |  |
| Bardach et al. (2016) | personal motivation |  |
| Bardach et al. (2016) | perceived confidence in the ability to make effective changes |  |
| Hung et al. (2019) | confidence in ability to engage in physical activities in difficult situations |  |
| Hung et al. (2019) | readiness to follow dietary advice |  |
| Rempe et al. (2019) | health and natural concerns |  |
| Rempe et al. (2019) | pleasure |  |
| Shatenstein et al. (2016)  Hung et al. (2019) | diet knowledge | Behavioral factors/skills  n=3 |
| Hung et al. (2019) | mobility |  |
| Hung et al. (2019) | meal preparation |  |
| Rempe et al. (2019) | long-term oriented eating style | Lifestyle practices  n=4 |
| Allès et al. (2016)  Andreeva et al. (2016) | smoking status |  |
| Granic et al. (2015)  Mumme et al. (2020) | physical activity |  |
| Mumme et al. (2020) | alcohol intake |  |
| Granic et al. (2015) | disability | Health status  n=10 |
| Host et al. (2016)  Oemichen and Smith (2016) | (physical) changes associated with aging |  |
| Watson et al. (2019) | dental status |  |
| Granic et al. (2015) | cognitive impairment |  |
| Granic et al. (2015) | depression |  |
| Granic et al. (2015)  Allès et al. (2016)  Andreeva et al. (2016) | body mass index |  |
| Andreeva et al. (2016) | hypertension |  |
| Shatenstein et al. (2016)  Rempe et al. (2019) | sensations of hunger/appetite |  |
| Hung et al. (2019) | risk of malnutrition |  |
| Hung et al. (2019) | oral-health related problems |  |
| Conklin et al. (2015) | double burden of low economic resources | Sociodemographic characteristics  n=8 |
| Atkins et al. (2015)  Dijkstra et al. (2015)  Kamphuis et al. (2015) | socioeconomic status |  |
| Dijkstra et al. (2015)  Granic et al. (2015)  Allès et al. (2016)  Andreeva et al. (2016)  Shatenstein et al. (2016)  Hung et al. (2019)  Van den Heuvel et al. (2019)  Mumme et al. (2020) | education |  |
| Andreeva et al. (2016)  Hung et al. (2019)  Van den Heuvel et al. (2019) | age |  |
| Conklin et al. (2015)  Andreeva et al. (2016)  Mumme et al. (2020) | gender |  |
| Atkins et al. (2015) | marital status |  |
| Hung et al. (2019) | financial difficulties |  |
| Atkins et al. (2015) | socio-economic factors in childhood |  |
| Oemichen and Smith (2016) | former experiences affecting eating behaviors | Previous life experiences  n=4 |
| Shatenstein et al. (2016) | diet quality change over time |  |
| Allès et al. (2016) | non-physical occupation over lifetime |  |
| Allès et al. (2016) | nutrient patterns over time |  |
| Dijkstra et al. (2015) | disliking fruits/vegetables | Others (individual level)  n=5 |
| Host et al. (2016) | personal resources |  |
| Hung et al. (2019) | being fussy about food |  |
| Rempe et al. (2019) | liking the food |  |
| Rugel and Carpiano (2015) | tangible and emotional/informational social support |  |
| **Social level** | | |
| Atkins et al. (2015) | adult living arrangements | Living arrangements (household)  n=3 |
| Dijkstra et al. (2015) | household income |  |
| Allès et al. (2016)  Andreeva et al. (2016)  Van den Heuvel et al. (2019)  Mumme et al. (2020) | living with others/alone |  |
| Conklin et al. (2015) | lack of social relationships | General social support  n=3 |
| Oemichen and Smith (2016) | social influences that play a role in decision making |  |
| Shatenstein et al. (2016) | social networks |  |
| **Physical level** | | |
| Kamphuis et al. (2015) | travel time to grocery shops | Geographical environment influence  n=4 |
| Andreeva et al. (2016) | residing in an urban/semi-urban area |  |
| Hawkesworth et al. (2017) | environment-diet relationships |  |
| Hung et al. (2019) | country |  |
| Sheats et al. (2017) | access to affordable healthy food and transportation | Food sources, access and availability  n=6 |
| Kamphuis et al. (2015) | available food being healthy |  |
| Kamphuis et al. (2015) | available food being tasteful |  |
| Oemichen and Smith (2016) | food access strategies: restaurants, retail markets, and alternative sources |  |
| Shanks et al. (2017) | support as a means of increasing food access |  |
| Shanks et al. (2017) | personal access to food sources |  |
| **Macro level** | | |
| Howell (2020) | “Sociocultural environment" (family influence, participation in social and cultural events) | Sociocultural complexity  n=1 |
| Rugel and Carpiano (2015) | programs/policies that seek to foster social support for older adults | Food policy  n=4 |
| Dijkstra et al. (2015)  Kamphuis et al. (2015) | cost of food |  |
| Rugel and Carpiano (2015) | ecologic interventions |  |
| Sheats et al. (2017) | involvement of older adults in policy discussion |  |
| Oemichen and Smith (2016) | financial and food insecurity driving use of food assistance programs | Food assistance programs  n=1 |
| Hawkesworth et al. (2017) | area income deprivation | Others (macro level)  n=2 |
| Sheats et al. (2017) | discovery tool to examine factors that facilitate or hinder access to food/food-related behaviors |  |
